# Assessing the Validity of the Ergotex IMU in Joint Angle Measurement: A Comparative Study with Optical Tracking Systems

**DOI:** 10.3390/s24061903

**Published:** 2024-03-16

**Authors:** Jose M. Jimenez-Olmedo, Juan Tortosa-Martínez, Juan M. Cortell-Tormo, Basilio Pueo

**Affiliations:** Health, Physical Activity, and Sports Technology Research Group, Faculty of Education, University of Alicante, 03690 San Vicente del Raspeig, Spain; j.olmedo@ua.es (J.M.J.-O.); juan.tortosa@ua.es (J.T.-M.); basilio@ua.es (B.P.)

**Keywords:** accelerometer, wearable, instrumentation, movement, kinematics

## Abstract

An observational, repeated measures design was used in this study to assess the validity of the Ergotex Inertial Measurement Unit (IMU) against a 3D motion capture system for measuring trunk, hip, and shoulder angles in ten healthy adult males (38.8 ± 7.3 y, bodyweight 79.2 ± 115.9 kg, body height 179.1 ± 8.1 cm). There were minimal systematic differences between the devices, with the most significant discrepancy being 1.4 degrees for the 80-degree target angle, denoting Ergotex’s precision in joint angle measurements. These results were statistically significant (*p* < 0.001), with predominantly trivial to small effect sizes, indicating high accuracy for clinical and biomechanical applications. Bland–Altman analysis showed Limits of Agreement (LoA) approximately ±2.5 degrees across all angles and positions, with overall LoA ranging from 3.6 to −2.4 degrees, reflecting Ergotex’s consistent performance. Regression analysis indicated uniform variance across measurements, with minor heteroscedastic errors producing a negligible underestimation trend of around 0.5 degrees in some instances. In conclusion, the Ergotex IMU is a reliable tool for accurate joint angle measurements. It offers a practical and cost-effective alternative to more complex systems, particularly in settings where precise measurement is essential.

## 1. Introduction

The conventional approach to motion analysis has traditionally been confined to controlled laboratory environments using motion-capture systems [1]. These systems ensure high-quality data and are considered the gold standard for movement quantification. However, these systems have some limitations, especially regarding their portability and the technical expertise required. These drawbacks limit their potential application in natural contexts such as clinical or sports settings [2]. To address these limitations, wearable sensors have been proposed due to their wireless nature, compact size, and lightweight design [3]. Wearable sensors have evolved considerably in recent years, including accelerometers and gyroscopes, known as inertial measurement units (IMUs) and, more recently, magnetic sensors [4]. This evolution has enabled their application in diverse contexts and settings, from assessing age-related kinematic changes to analyzing the biomechanics of specific sports movements in natural contexts [5,6]. Furthermore, their wireless and portable nature helps quantify body movements over extended periods during functional tasks and benefits, for example, physical rehabilitation assessments [7].

In this sense, assessing different movements’ range of motion is of particular interest; for example, to prevent or rehabilitate injuries as well as to analyze sports performance. A systematic review conducted in 2010 [8] proposed IMUs as a reliable tool for assessing range of motion, with subsequent reviews emphasizing the influence of joint, task, and data processing techniques on accuracy [2,9]. Investigations into various body regions for validity and/or reliability have been conducted, revealing IMUs as a viable alternative to motion-capture systems [2,7]. They particularly excel in capturing flexion/extension movements in lower limb joints during simple activities.

However, inertial sensors also have limitations, such as limited accuracy, calibration requirements, sensor drift, and a restricted range of motion [7]. Notwithstanding, IMU validity varies with task complexity, showing higher accuracy in simpler tasks and improved precision for lower limb joints. Thus, as movement complexity increases, validity tends to decrease, as observed in assessments involving sports-related tasks or daily activities such as table sweeping. Proper calibration, including precise alignment with anatomical segment axes, is pivotal to heightened reliability in these measurements [2]. These calibration procedures demand meticulousness and can be time-consuming, requiring specialized expertise. Furthermore, sensor drift over time impacts accuracy, and the limited range of motion may restrict their use in specific populations or movements [7].

Since numerous commercial and homemade IMU systems are available for research and clinical applications, the need to ensure their effectiveness, particularly in rehabilitation programs, fall detection systems, and personalized healthcare monitoring devices is crucial [10]. Before field implementation, validation against a reliable reference is crucial. Further research is needed to establish the validity, reliability, and sensitivity to changes in wearable sensors for measuring the range of motion. Standardization of calibration and filtering methods is vital to improving accuracy and consistency across different populations and measurement protocols [7].

The IMU presented in this study originated from the Lumbatex project, which aims to integrate IMUs into textile garments for monitoring spinal curvature and motion [11]. The refinement of this technology led to the Ergotex IMU (JVTech Solutions, Alicante, Spain), which eliminates noise associated with textile garments and incorporates specific calibration protocols and sensor placement. Ergotex, designed for smartphones or tablets, is compact and tailored for lumbar or pelvic kinematic analysis. It has recently expanded to other joints, including shoulder, trunk, and hip flexion/extension. This technology aligns with rehabilitation medicine trends toward mHealth applications, which have shown versatility in various rehabilitation contexts [12]. Although the manufacturer’s specifications highlight Ergotex’s capability to monitor joint position with multiple sensors, there is currently no validation study assessing this device’s accuracy for multiple joints. Hence, this study quantifies the concurrent validity of Ergotex in measuring trunk, hip, and shoulder angles in healthy adults. These measurements are then compared to a 3D motion capture system, which is considered the gold standard for analyzing human movement kinematics.

## 2. Materials and Methods

### 2.1. Study Design

This study adopted an observational design to evaluate the concurrent validity of ROM measurements for the trunk, hip, and shoulder joints. This methodology involved placing sensors in three distinct locations: the lower back and legs for the hip joints, and the arms for the shoulder joints. The primary objective was to compare data obtained from the Ergotex IMU against those from a laboratory-standard optical tracking system designated as the criterion instrument.

To ensure statistical robustness, the study was designed to achieve 95% statistical power. Using G*Power software (version 3.1.9.7, Heinrich-Heine-Universität Düsseldorf, Düsseldorf, Germany), a minimum sample size of 45 concurrent measurements was established. This calculation was based on an alpha level of 0.1 for a two-tailed test. To meet this sample size requirement, each of the 10 participants performed five repetitions of each target angle in all three sensor positions (lower back, leg, and arm). This approach produced a comprehensive dataset of 950 concurrent measurements.

### 2.2. Instrumentation

#### 2.2.1. Ergotex

The Ergotex device (JVTech Solutions, Alicante, Spain) integrates a triaxial accelerometer, a gyroscope, and a magnetometer. Its specifications include a weight of 8 g and dimensions of 23 × 21 × 10 mm. Designed for a sampling rate of 20 Hz, the Ergotex can be attached to the skin with double-sided adhesive tape or secured with an elastic band in other locations. The Ergotex captures acceleration in three dimensions, processes the acceleration signal internally, and transmits the data in real-time via Bluetooth to a compatible smartphone or tablet running the Ergotex application. This app provides immediate visualization of the data and allows exporting to CSV format. Calibration of the Ergotex is necessary before each use, ensuring accuracy across all axes. During the calibration procedure, the device is placed on a level surface so the software can identify each of the octahedron’s six faces and set up the IMU’s local 3D coordinate system. The device is powered on five minutes prior to testing and outfitting the subjects. A static trial, consisting of a 10 s ground placement of the device, precedes actual testing and is used to estimate and adjust the gyroscope’s bias [13].

#### 2.2.2. Optical Tracking System

The optical tracking system (OptiTrack, NaturalPoint Inc., Corvallis, OR, USA) is designed for real-time assessment of the position and orientation (six degrees of freedom) of rigid bodies. These bodies contain a minimum of three optical markers that maintain consistent distances and angles among themselves, ensuring stable spatial properties. These optical markers, passive spheres with retro-reflective surfaces, are engineered to reflect infrared light directly back to its source. To capture this reflection, eight OptiTrack Flex:v100R2 cameras arranged around the monitoring area were used. These cameras, each featuring a VGA resolution of 640 × 480 pixels and capable of 100 frames per second, emit infrared light through a ring of 26 LEDs (IR 850 nm) that are synchronized with their capture shutters. Their operation at 100 Hz and a shutter speed of 20 µs achieve a global resolution of 0.001 m, providing detailed and accurate data over a considerable working volume, thus fitting our study requirements.

Data acquisition and processing were managed by Tracking Tools software v. 2.5.3 (OptiTrack, NaturalPoint Inc., Corvallis, OR, USA). This software computes the position and orientation (pose) of any rigid body within the monitored space. Prior to data collection, calibration of both extrinsic (physical position and orientation) and intrinsic (focal length and lens distortion) camera parameters was conducted. This calibration involved the use of a three-marker OptiWand and a specific algorithm within Tracking Tools software [14,15]. The gathered data was relayed through a USB connection to a laptop and analyzed with Motive Tracker 2 software (OptiTrack, NaturalPoint Inc., Corvallis, OR, USA). This software not only ensures synchronization and calibration of image collection systems but also facilitates data exportation in CSV format for further analysis in spreadsheets, specifically to determine rigid body orientation.

**Figure 1:**
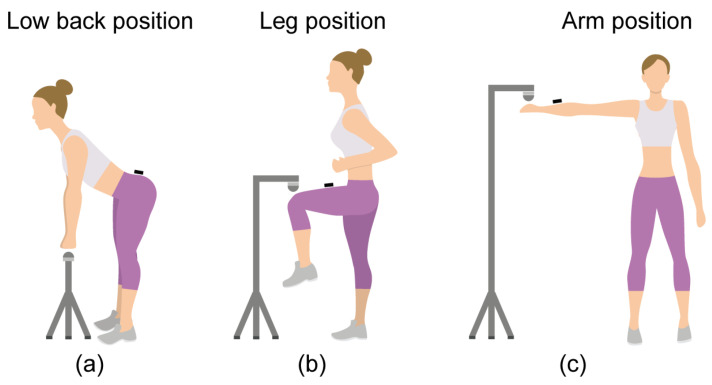
Illustration of Movement Protocols. (**a**) Hip flexion with sensor placement on the lower back; (**b**) hip flexion with sensor attached to the mid-thigh position; (**c**) shoulder abduction with sensor placement on the upper arm.

To establish the reference standard orientation of the Ergotex, a rigid body instrumented with four spherical optical markers (1.1 cm diameter, Hand Rigid Body, NaturalPoint Inc.) was attached to the Ergotex sensor. These markers, arranged in a coplanar fashion around the sensor, help determine its precise orientation. The selection of this method as the gold standard is attributed to the optical system’s high accuracy in marker position measurement [8]. The markers were meticulously affixed to rigid plastic boards to minimize skin movement artifacts [16]. In this study, the Ergotex, along with the optical’s rigid body, was positioned on the sacrum, atop the pelvic segment, as depicted in Figure 1.

### 2.3. Procedure

The experimental procedure was conducted in a single session at the Movement Analysis Laboratory at the University of Alicante. Initially, participants were introduced to experimental protocols and underwent anthropometric measurements for familiarization purposes. The sensor placement was strategically chosen to assess hip motion in the sagittal plane and shoulder motion in the frontal plane, guided by clinical expertise. Participants were instructed to perform hip and shoulder movements, ensuring natural and controlled motion, to accurately capture a joint range of motion. The orientation and placement of the rigid body and IMU were uniform across participants. After familiarization, each participant performed five repetitions of each movement at specified target angles. For hip flexion ROM in the lower back position, the Ergotex and rigid body were securely attached over the sacral bone of S2, employing a palpation technique [17,18]. Participants began in an upright stance with extended legs and feet hip-width apart. They then flexed the hip joint while maintaining a straight back, reaching predetermined target angles from 10 to 70 degrees in 10-degree increments, as shown in Figure 1a. Each angle was replicated five times, aided by an adjustable support piece set at hip height for each target angle. For hip flexion ROM in the leg position, the devices were affixed to the middle lateral thigh, equidistant between the hip and knee joints [19]. Participants stood with feet shoulder-width apart, hands on hips, and executed hip flexion aligned with reference points representing target angles from 10 to 70 degrees in 10-degree steps, as depicted in Figure 1b. This movement was controlled and deliberate, with each angle repeated five times. Finally, for shoulder abduction ROM, the sensor attachment was affixed to the forearm, midway between the olecranon and radial styloid, using a self-adhering strap [20]. Participants, in an upright position with arms relaxed, lifted their arms laterally to designated target angles of 20, 40, 60, 80, and 100 degrees, as shown in Figure 1b. An adjustable element at shoulder height ensured consistent angle measurement for each target. Each angle was performed five times for measurement consistency. This procedure yielded 35 measurements each for the trunk and leg, and 25 for the arm per participant. These measurements were recorded concurrently by both the Ergotex and the optical tracking system.

### 2.4. Participants

Ten healthy male adults participated in this study (38.8 ± 7.3 year, bodyweight 79.2 ± 115.9 kg, body height 179.1 ± 8.1 cm). Criteria for participation included no diagnosed musculoskeletal or neurological conditions impacting the lower back or pelvis, absence of recent lower back or pelvic surgery or injuries in the preceding six months, and the ability to comprehend and execute the required movements during the experiment. The study was conducted following the ethical principles of the Declaration of Helsinki. Before the study commenced, the participants provided their informed written consent. This research received approval from the University of Alicante’s Ethics Committee (protocol code UA-2023-11-16).

### 2.5. Statistical Analysis

Data are represented using descriptive statistics, including mean and standard deviation (SD), along with 95% confidence intervals (CI). A paired samples *t*-test was used to assess systematic differences between the Ergotex and the criterion instrument. The magnitude of these differences was quantified using the bias-adjusted Hedges’ effect size (ES), according to Hopkins et al. [21]: trivial (less than 0.2), small (0.2 to 0.6), moderate (0.6 to 1.2), and large (greater than 1.2).

Bland–Altman plots were employed to assess the concordance between the two instruments, displaying the average of outcomes against their differences to spot random errors and proportional biases. Homoscedasticity of errors was identified if the slope of the regression line and Pearson’s correlation coefficient (*r*) of differences against the mean were significantly different from zero (*p* > 0.05) [22]. Additionally, Pearson’s product–moment correlation coefficient (*r*) with a 95% CI was calculated to further analyze the relationship between the two instruments. The interpretation thresholds were set to trivial (below 0.1), small (0.1 to 0.3), moderate (0.3 to 0.5), high (0.5 to 0.7), very high (0.7 to 0.9), and practically perfect (above 0.9), according to [23].

Ordinary least square (OLS) linear regression analysis was conducted to explore the relationship between paired data from the instruments, as expressed through the equation *y* = *β*_1_*x* + *β*_0_, where *y* is the dependent variable and *x* is the independent variable. The slope *β*_1_ indicates proportional differences, while the intercept *β*_0_ reflects systematic differences quantitatively. The standard error of estimate (SEE), calculated in both raw and standardized units [24], was interpreted using thresholds adapted from Cohen’s scale [23]: trivial (below 0.1), small (0.1 to 0.3), moderate (0.3 to 0.6), large (0.6 to 1.0), very large (1.0 to 2.0), and extremely large (above 2.0). Lower SEE values signify less estimation error, indicating a closer fit to the regression line. Statistical analyses were performed using IBM SPSS v. 22 (IBM Corp, Armonk, NY) and a spreadsheet specifically designed for validity assessments [25].

## 3. Results

Table 1 presents the mean values for each target angle, sensor position, and overall condition. The systematic differences between the devices were minimal, with maximum values of 1.4 degrees for the 80-degree target angle, 1.2 degrees for the leg position, and 0.5 degrees overall. All differences were statistically significant at *p* < 0.001. The practical significance of these maximum values indicates a moderate effect size (ES = 0.65) for the target angle and a trivial effect (ES = 0.06, 0.02) for the leg position and overall conditions. The remaining comparisons showed biases of approximately 0.5 degrees with a trivial effect, suggesting that the Ergotex offers more consistent outcomes than the gold standard optical tracking system.

**Figure 2:**
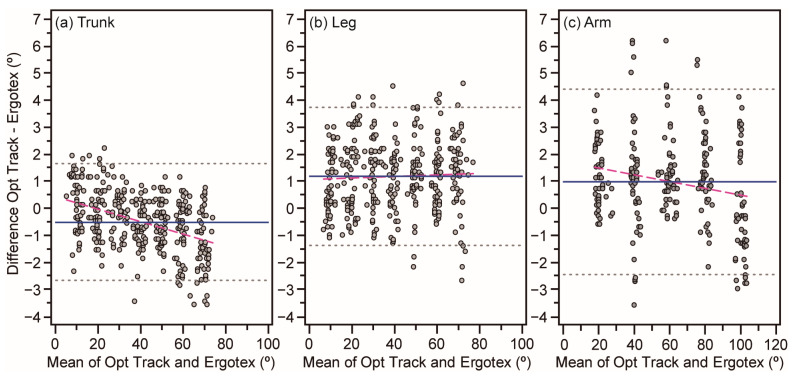
Assessment of agreement between joint angle measurements from the optical tracking and Ergotex systems for trunk (**a**), leg (**b**), and arm (**c**) positions. The solid blue line represents the average systematic bias or the mean difference in measurements between the two devices. The upper and lower dotted lines delineate the 95% limits of agreement (mean ± 1.96·SD), indicating the expected range of random measurement error. The pink dashed line illustrates the linear regression of mean differences, providing insight into proportional bias.

**Figure 3:**
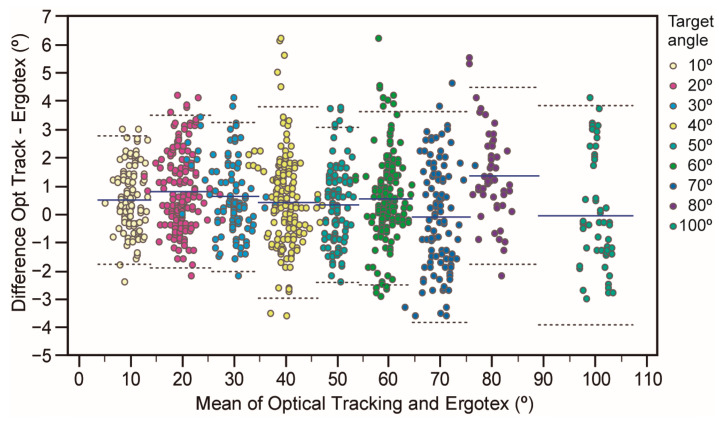
Assessment of agreement between joint angle measurements from the optical tracking and Ergotex systems for all target angles. The solid blue lines represent the average systematic bias or the mean difference in measurements between the two devices. The upper and lower dotted lines delineate the 95% limits of agreement (mean ± 1.96·SD), indicating the expected range of random measurement error.

Figure 2 and Figure 3 illustrate the concordance of angle measurements between the optical tracking criterion device and the Ergotex IMU for the three sensor positions and the target angle, respectively. Both figures demonstrate a high degree of agreement between the devices, as most data points fell within the ±95% LoA. The dashed lines in the Bland–Altman plots reveal a slight degree of heteroscedasticity for the lower back and arm positions, as well as for the 30, 40, and 80-degree angles. However, the impact is minor, confirming that Ergotex can produce proportional errors across the measurement range.

Table 1 also details the numerical outcomes of the Bland–Altman analysis. The LoA is approximately ±2.5 degrees for all target angles, sensor positions, and overall conditions. Angles exhibiting the broadest LoA are 70 degrees (from 3.6 to −3.8 degrees) and 100 degrees (from 3.8 to −2.9 degrees), yet their biases are almost negligible (−0.04 and −0.01 degrees, respectively). Concerning sensor positions, the arm position has the widest LoA (from 4.4 to −2.4 degrees), with a bias of 0.99 degrees. The overall LoA span was from 3.6 to −2.4 degrees, with a 0.5 degree bias.

Regarding proportional bias assessment across the range of instrument measurements, regression analysis of the paired differences against the means yielded slope values close to zero for all conditions (target angle, position, and overall), as depicted in Table 1. These findings indicate that the Ergotex produces homoscedastic errors across all conditions, ensuring uniform variance across a range of measurements. However, there were three instances where both the slope and Pearson’s correlation coefficient were not significantly different from zero for specific target angles: 30 degrees (slope = −0.12, *r* = −0.28, *p* = 0.005), 40 degrees (slope = −0.16, *r* = −0.22, *p* = 0.007), and 80 degrees (slope = −0.41, *r* = −0.51, *p* = 0.0002). These heteroscedastic errors suggest that the Ergotex produces a slight underestimation of approximately 0.5 degrees as the measurement range increases. A similar trend was observed in measurements of the lower back (slope = −0.16, *r* = −0.22, *p* = 0.007) and arm positions.

**Figure 4:**
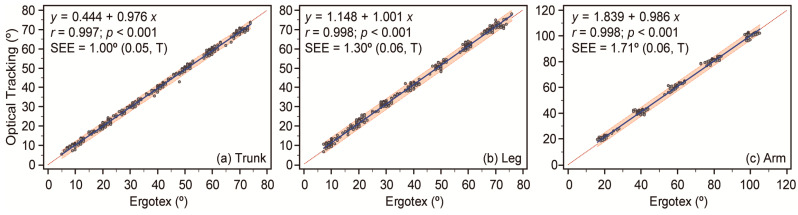
Correlation of angle measurements between optical tracking and the Ergotex for trunk (**a**), leg (**b**), and arm (**c**) positions. The solid blue line represents the linear regression trend, while the dotted line indicates the line of equality (*y* = *x*) for comparison. The orange-shaded region represents the 95% prediction interval and visualizes the expected measurement variance. Statistical indicators, such as Pearson’s correlation coefficient (*r*), its statistical significance (*p*), and the Standard Error of Estimate (SEE) are provided, with the SEE’s practical significance denoted as trivial (T). Each point represents a concurrent measurement across both devices, offering a comparative analysis of their performance.

Figure 4 illustrates the linear relationship between optical tracking and the Ergotex across three sensor positions. The slope of the fitted OLS equations approximated unity for all positions: 0.976 [0.970; 0.981] for the trunk, 1.001 [0.994 to 1.007] for the leg, and 0.986 [0.978; 0.993] for arm positions, all *p* < 0.001. These values close to unity indicate a strong linear agreement between optical tracking and Ergotex measurements, suggesting that Ergotex is a reliable tool for capturing movement angles comparable to the established optical tracking system. The SEE was low, indicating high precision in the measurements: 1.00 [0.93; 1.08] deg for the lower back, 1.30 [1.22; 1.41] deg for the leg, and 1.71 [1.57; 1.87] deg for the arm. These values were assessed as trivial according to the standardized SEE of 0.05 [0.05; 0.06], 0.06 [0.06; 0.07], and 0.06 [0.05; 0.07] for the lower back, leg, and arm, respectively. The low SEE across all positions reinforces the Ergotex’s accuracy in capturing movement angles, further supporting its utility in clinical and research settings where precise angle measurements are essential.

## 4. Discussion

This study quantified the concurrent validity of Ergotex by measuring trunk, hip, and shoulder angles in healthy adults and compared these measurements to a 3D motion capture system. Our results indicate that the Ergotex, when compared to the gold standard optical tracking system, demonstrates remarkable accuracy in measuring joint angles across various conditions and sensor placements. The maximum systematic differences observed are minimal, with the largest being 1.4 degrees for the 80-degree target angle. Statistically significant differences (*p* < 0.001) confirm the reliability of these findings. The practical significance of different target angles provides a nuanced picture of the Ergotex’s accuracy. Although the peak effect size reached a moderate level of 0.64 for a target angle of 80 degrees, indicating the device’s potential utility in applications demanding precise angle measurements, such as biomechanical studies or clinical assessments, this value is an outlier. For most of the target angles evaluated, effect sizes were observed to be small (0.22, 0.33, 0.28) or trivial (0.19, 0.18, 0.18, 0.04, 0.01). Therefore, the Ergotex’s overall utility should be considered within the context of predominantly trivial to small effect sizes, with its accuracy being most pronounced at a singular target angle. This finding suggests that the Ergotex is sufficient for applications where precise angle measurement is critical (such as biomechanical studies or clinical assessments).

For the leg position and overall conditions, the trivial effect sizes (ES = 0.06, 0.02) imply that the differences, though statistically significant, may not be practically meaningful in these contexts. For the remaining comparisons, the biases were approximately 0.5 degrees with a trivial effect, reinforcing Ergotex’s potential to deliver consistent and reliable outcomes in various settings. This consistency is particularly valuable in clinical and research environments, where measurement precision is essential for accurate diagnosis, treatment planning, and scientific analysis. Overall, these findings suggest that the Ergotex is a viable tool for accurate joint angle measurements. It offers a practical alternative to more complex and expensive systems without significant compromise on measurement accuracy.

Most studies have analyzed the validity and/or reliability of different IMUs for measuring joint motion during several movements and/or activities [2,7,26,27]. However, there is a broad consensus that the more complex the joint motion analyzed (e.g., involving more anatomical planes, higher intensities/speeds/accelerations during movement, etc.), the less valid and reliable IMUs are for this purpose [2,7]. For this reason, in our study, simple and controlled joint movements were performed, so that our results could be compared to studies of a similar nature (e.g., isolated and controlled joint motion). In this regard, our results show similar systematic errors to those reported in similar studies [28,29,30,31,32].

The error range published by Mjøsund et al. [30] when quantifying trunk movement against the Vicon optical tracking system falls within similar values to our study, between 0.71 and 1.82 degrees. Similarly, Bauer et al. [32] reported error ranges between 1.1 and 6.8 degrees for hip motion and between 2.6 and 5.9 degrees for trunk motion after comparing IMUs to the Vicon optical tracking system. Compared to the Ariel Performance Analysis System, a video-based 3D motion analysis system using Direct linear transformation (DLT) algorithms, the error range for IMUs in shoulder movement was between 0.63 and 1.98 degrees [28]. Similarly, Cutti et al. [29] found error values between 0.2 and 3.2 degrees for shoulder motion against the Vicon system. Likewise, Robert-Lachaine et al. [31] identified error values ranging from 1.3 to 3.6 degrees for isolated hip, trunk, and shoulder motions compared to the OptiTrack system, which was used in our study.

Only three studies focused on shoulder motion reported higher error values of up to 20 degrees [33,34,35]. The first study reported mean peak differences of up to 17.25 degrees in shoulder movement when comparing IMUs to the Smart-DX 7000 optical tracking system [35]. Secondly, Bouvier et al. [33] reported error values of up to 19.3 in shoulder motion against the Eagle 4 video-based 3D motion analysis system. Finally, Kumar et al. indicated the largest error values quantifying shoulder and hip angles (between approximately 10 and 30 degrees) [34], which were attributed to the use of goniometry as the gold standard.

In our study, findings from Figure 2 and Figure 3 along with the Bland–Altman analysis offer a detailed perspective on the concordance between the Ergotex IMU and the optical tracking criterion device. The high degree of agreement noted in most data points within the ±95% Limits of Agreement (LoA) underscores Ergotex’s reliability in measuring angles accurately across various conditions. The presence of slight heteroscedasticity in certain sensor positions and angles, while notable, does not significantly detract from the overall performance of the Ergotex. Numerical outcomes from the Bland–Altman analysis reinforce this, with the LoA for all target angles, sensor positions, and overall conditions being approximately ±2.5 degrees. This range is acceptable for many practical applications, especially considering the negligible biases observed in most measurements. Broader LoA at certain angles and the arm position could suggest areas where Ergotex accuracy slightly varies. Despite minor biases associated with these measurements, the Ergotex remains a reliable tool for angle measurement in these contexts. Its consistency across a range of angles and positions makes the Ergotex a versatile and dependable option in both clinical and research settings, where precise angle measurements are crucial.

Figure 4’s data and associated analysis provide compelling evidence of Ergotex’s efficacy in angle measurement. The slopes of the fitted Ordinary Least Squares (OLS) equations for all sensor positions being close to unity, along with statistically significant *p*-values, demonstrate a strong linear agreement between the Ergotex and optical tracking system. This finding indicates that Ergotex can reliably replicate established system measurements. Moreover, the Standard Error of Estimate (SEE) values across all positions are low, indicating high measurement precision. The trivial standardized SEE values reinforce this precision, highlighting the Ergotex’s ability to capture movement angles with high accuracy. This level of precision and reliability in angle measurement makes the Ergotex a valuable tool for both clinical and research settings where accurate and consistent angle measurements are crucial for effective diagnosis, treatment, and scientific investigation.

Our findings are consistent with earlier studies that used Bland–Altman analysis [28,30,31,34], indicating that most data points fall within two standard deviations of error. In this sense, Mjøsund et al. [30] reported 95% LoA ranging from 4.7 to −3.9 degrees. Robert-Lacheine et al. [31] reported a low bias of around 1 degree, with a repeatability coefficient ranging from 2.2 to 9.7 degrees. Notably, our results align with those reported by other authors for linear regression or correlation analysis [29,31,32,33,34,35]. The correlation coefficients ranged between 0.9 [31,35], above 0.9 [33], and 0.99 [29]. Similarly, Bauer et al. [32] reported coefficients of determination between 0.85 and 0.99, corresponding to correlation coefficients of 0.92 to 0.99.

This study, while robust in its findings, has certain limitations. Firstly, comparing only one alternative device (Ergotex) to the gold standard may limit the generalizability of the results. Additionally, the study’s focus on specific sensor positions and target angles may not fully represent the devices’ other potential uses in different anatomical areas or activities. Furthermore, the environmental conditions under which the measurements were obtained were controlled and may not reflect real-world scenarios where factors such as movement speed and external interferences can affect device accuracy. Lastly, the study’s sample size and demographic may limit its applicability across a wider population. Notwithstanding these limitations, future research should include the evaluation of devices, such as the Ergotex IMU, over prolonged periods. These measures allow for a more comprehensive assessment of their durability and measurement consistency under varying conditions.

## 5. Conclusions

This research compared Ergotex’s validity to an optical tracking system for measuring trunk, hip, and shoulder angles. The Ergotex showed consistent and accurate estimations with an average bias of approximately 0.5 degrees, reinforcing its ability to provide reliable outcomes across various sensor positions and movement types. Negligible deviations observed through the SEE further confirm the precision and reliability of both the Ergotex and optical tracking measurements.

These findings collectively confirm the effectiveness of the Ergotex in providing accurate estimations of trunk, hip, and shoulder parameters. The implications for clinical practice are substantial, positioning the Ergotex as a promising alternative to laboratory-based instruments for estimating these parameters. However, further research with larger and more diverse samples is necessary to reinforce these conclusions and expand Ergotex’s applicability in clinical settings.

## Figures and Tables

**Table 1:** Mean outcomes from optical tracking and Ergotex, comparison of bias, limits of agreement, interval estimates, and regression parameters for target angles, sensor position, and overall conditions derived from Bland–Altman analysis.

	Opt	Ergo	Bias	ES	+LoA	−LoA	Int	Slope	*r*	*p*
10 deg	10.3	9.8	0.51 ***	0.22 S	2.79	−1.77	0.24	0.02	0.04	0.72
+95%CI	10.7	10.2	0.74	0.50 S	3.19	−1.37	3.19	1.73	0.23	
−95%CI	10.0	9.6	0.28	−0.06 T	2.40	−2.16	2.40	−1.25	−0.16	
20 deg	20.3	19.6	0.79 ***	0.33 S	3.49	−1.90	1.07	−0.01	−0.02	0.78
+95%CI	20.7	19.9	0.57	0.56 S	3.88	−1.53	3.00	0.08	0.14	
−95%CI	20.0	19.1	1.01	0.10 T	3.11	−2.29	−0.86	−0.11	−0.19	
30 deg	29.8	29.2	0.62 ***	0.19 T	3.25	−2.00	4.19	−0.12	−0.28	<0.01
+95%CI	30.4	29.9	0.89	0.47 S	3.71	−1.55	6.65	−0.04	−0.09	
−95%CI	29.2	28.5	0.36	−0.08 T	2.79	−2.46	1.73	−0.20	−0.45	
40 deg	40.3	39.9	0.43 **	0.18 T	3.82	−2.96	7.01	−0.16	−0.22	<0.05
+95%CI	40.7	40.3	0.71	0.41 S	4.29	−2.48	11.80	−0.04	−0.06	
−95%CI	39.9	39.5	0.15	−0.05 T	3.34	−3.44	2.23	−0.28	−0.37	
50 deg	50.2	49.8	0.34 *	0.18 T	3.08	−2.40	−6.68	0.14	0.14	0.17
+95%CI	50.5	50.1	0.62	0.46 S	3.56	−1.92	3.45	0.34	0.32	
−95%CI	49.9	49.6	0.06	−0.10 T	2.61	−2.87	−16.81	−0.06	−0.01	
60 deg	60.1	59.6	0.56 ***	0.28 S	3.62	−2.50	−5.16	0.09	0.11	0.17
+95%CI	60.5	59.9	0.81	0.51 S	4.06	−2.07	3.07	0.23	0.27	
−95%CI	59.8	59.3	0.31	0.05 T	3.19	−2.93	−13.40	−0.04	−0.05	
70 deg	69.7	69.8	−0.10	−0.04 T	3.63	−3.83	−3.95	0.05	0.06	0.55
+95%CI	70.2	70.3	0.27	0.23 S	4.28	−3.18	8.67	0.23	0.25	
−95%CI	69.3	69.4	−0.47	−0.32 S	2.98	−4.48	−16.57	−0.12	−0.14	
80 deg	80.7	79.3	1.37 ***	0.64 M	4.50	−1.74	33.91	−0.41	−0.51	<0.01
+95%CI	81.2	80.0	1.83	1.04 M	5.28	−0.97	49.93	−0.21	−0.27	
−95%CI	80.2	78.6	0.92	0.23 S	3.72	−3.91	17.88	−0.61	−0.68	
100 deg	100.6	100.6	−0.03	−0.01 T	3.84	−2.93	29.57	−0.29	−0.27	0.06
+95%CI	101.1	101.3	0.53	0.38 S	4.81	−4.87	60.00	0.01	0.01	
−95%CI	100.1	99.9	−0.59	−0.41 S	2.88	−0.00	−0.86	−0.59	−0.51	
Trunk	39.8	40.4	−0.54 ***	−0.02 T	1.65	−2.71	0.39	−0.023	−0.41	<0.01
+95%CI	41.9	42.5	−0.42	0.13 T	1.84	−2.51	0.64	−0.017	−0.32	
−95%CI	37.8	38.3	−0.65	−0.16 T	1.44	−2.91	0.16	−0.028	−0.50	
Leg	40.1	38.9	1.17 ***	0.06 T	3.73	−1.38	1.06	0.003	0.05	0.41
+95%CI	42.3	41.1	1.31	0.21 S	3.96	−1.14	1.36	0.009	0.15	
−95%CI	38.0	36.8	1.04	−0.14 T	3.49	−1.61	0.77	−0.004	−0.06	
Arm	60.8	59.8	0.99 ***	0.03 T	4.42	−2.44	1.74	−0.012	−0.20	<0.01
+95%CI	64.3	63.3	1.21	0.21 S	4.79	−2.06	2.24	−0.005	−0.08	
−95%CI	57.3	56.2	0.77	−0.14 T	4.05	−2.81	1.24	−0.020	−0.32	
Overall	45.5	45.0	0.50 ***	0.02 T	3.60	−2.60	0.74	−0.005	−0.08	<0.05
+95%CI	47.0	46.5	0.59	0.11 T	3.78	−2.43	0.94	−0.001	−0.02	
−95%CI	43.9	43.4	0.39	−0.07 T	3.43	−2.78	0.52	−0.009	−0.14	

* *p* < 0.05, ** *p* < 0.001, *** *p* < 0.0001. Opt (optical tracking), Ergo (Ergotex), Bias, LoA (limits of agreement), Int (intercept) is expressed in degrees, and slope is expressed in degrees/degrees. ES: Effect Size (T: trivial, S: small, M: moderate); *r*: Pearson’s product–moment correlation coefficient of the differences against the means; *p*: statistical significance of the slope coefficient in the regression analysis; CI: confidence interval.

## Data Availability

The data that support the findings of this study are available from the corresponding author upon reasonable request.
